# Subsegmentectomy versus segmentectomy resection for the treatment of operable patients with stage IA non-small cell lung cancer: A meta-analysis

**DOI:** 10.3389/fsurg.2022.1060507

**Published:** 2023-01-06

**Authors:** Liwei Song, Shuping Li, Xuefeng Hao, Renjing Jin, Wei Li, Minghang Zhang, Jinghui Wang, Shijie Zhou, Teng Ma, Shaofa Xu

**Affiliations:** ^1^ Department of Thoracic Surgery, Beijing Chest Hospital, Capital Medical University, Beijing Tuberculosis and Thoracic Tumor Research Institute, Beijing, China; ^2^ Cancer Research Center, Beijing Chest Hospital, Capital Medical University, Beijing Tuberculosis and Thoracic Tumor Research Institute, Beijing, China; ^3^ Heart Center, Beijing Tuberculosis and Thoracic Tumor Research Institute, Beijing Chest Hospital, Capital Medical University, Beijing, China; ^4^ Department of Thoracic Surgery, Beijing Tiantan Hospital, Capital Medical University, Beijing, China

**Keywords:** non-small cell lung cancer, segmentectomy, subsegmentectomy, meta-analysis, safety

## Abstract

**Background:**

There were new points of interest in performing subsegmentectomy and segmentectomy for patients with early stage non-small cell lung cancer (NSCLC). However, whether patients who underwent subsegmentectomy could obtain satisfactory clinical outcomes remains unclear. The present study aimed to compare the clinical outcomes and security of surgical procedures between subsegmentectomy and segmentectomy.

**Methods:**

A systematic review and meta-analysis was performed through five online databases to identify the included literatures which presented intact clinical outcome data among different surgical procedures. The included studies were evaluated based on precise and predefined inclusion criteria.

**Results:**

There were 4 published studies identified in this meta-analysis. A total of 325 patients who underwent subsegmentectomy and 904 patients who underwent segmentectomy were involved in this analysis. The duration of drainage [MD −0.19; 95%CI (−0.36, −0.02), *p *= 0.03] and postoperative hospital stay [MD −0.30; 95%CI (−0.58, −0.02), *p *= 0.009] of subsegmentectomy were significantly less than that of segmentectomy. There was no statistically significant difference among recurrence rate [OR 0.85; 95%CI (0.21, 3.42), *p* = 0.82], operation time, blood loss, incidence of complications [OR 0.83; 95%CI (0.58, 1.20), *p* = 0.33] between subsegmentectomy and segmentectomy in patients with stage IA NSCLC.

**Conclusion:**

The meta-analysis was firstly performed to compare perioperative outcomes among surgical procedures. The perioperative outcomes were comparable between subsegmentectomy and segmentectomy. Subsegmentectomy might be an alternative treatment for the deep tumor with size less than 1.5 cm and mainly composed of Ground Glass Opacity (GGO).

## Introduction

In the past decades, anatomical lobectomy has been considered to be the standard surgical treatment for early stage NSCLC (1). Widespread application of high resolution computed tomography (HRCT) and popularization of lung cancer screening have promoted the detection of early stage NSCLC (2). Previous published study reported that patients underwent sublobar resection had a significantly higher rate of recurrence than those in lobectomy (3). The reason for this result may be that most patients enrolled in the study underwent wedge resection not segmentectomy. We deduced the increased local recurrence following wedge resection was partly caused by the inadequate resection margin. Wedge resection was reported as a risk factor for local recurrence (4–6). With the recent advancements of three-dimensional computed tomography (3D-CT) simulation and surgical techniques, segmentectomy becomes more general in clinical practice and may have comparable outcome compared with lobectomy (7–11). Phase 3, randomized, controlled trials of patients with small peripheral lung cancers, including CALBG 140503 and JCOG0802/WJOG4607L, are ongoing, and the validated non-inferiority of sublobar resections was recently reported (9, 12). The trial JCOG0802 suggested that segmentectomy should be the standard surgical procedure for patients with small-sized peripheral NSCLC (9). In order to radically dissect the tumor, avoid excessive resection and preserve more pulmonary function, some researchers carried out thoracoscopic subsegmentectomy, which represents smaller resection scope than segmentectomy (13, 14). Subsegmentectomy is performed with the assistance of three-dimensional computed tomography bronchography and angiography (3D-CTBA) and supported by superb surgical techniques (15). Although recent studies showed that subsegmentectomy had comparable outcomes compared with segmentectomy in small sized NSCLC (16), whether patients with early stage benefit from subsegmentectomy still remains controversial. There is a lack of sufficient evidence regarding the clinical outcome of subsegmentectomy compared with segmentectomy. Up to now, the indication of subsegmentectomy is not well clear. This analysis aimed to compare perioperative outcomes and security for patients who underwent either subsegmentectomy or segmentectomy.

## Patients and methods

### Study search strategy

On February, 2022, we searched for relevant studies as following databases: Pubmed, Embase, Web of science, China National Knowledge Infrastructure (CNKI) and Wanfang. To identify all relevant studies, we combined search terms (“Non-Small Cell Lung Cancer” or “NSCLC” or “Non-Small Cell Lung Carcinoma”) and (“Subsegmentectomy”) and (“Segmentectomy” or “Segmentectomies”) with the Boolean Operators “AND” or “OR”. To identify high quality of studies, we prespecified the inclusion and exclusion criteria.

### Selection criteria

Eligible studies that reported recurrence rate, operation time, blood loss, postoperative hospital stay, duration of drainage and complications were included. The final results were given an appraisal based on the included and excluded criteria.

We chose the eligible studies as following inclusion criteria and exclusion criteria:

Inclusion criteria:

(1) Trials in which patients underwent segmentectomy or subsegmentectomy for early stage NSCLC;
(2) Trials in which comparative perioperative outcomes were analyzed;

Exclusion criteria:

(1) Patients who suffered from tumors of other organs simultaneously;
(2) Abstracts, case report, conference presentations and expert opinions were excluded.

### Study appraisal

The two investigators extracted the following data: the publication year, first author, study design, study period, number of participants, characteristics of participants. The investigators were required to evaluate the quality of identified studies independently. Discrepancies between the two reviewers were resolved by discussion and consensus. The final results were decided by the senior investigator. The methodology evaluation of this study was conducted by the Newcastle-Ottawa Scale (NOS) (17).

### Statistical analysis

Meta-analysis was performed by combining the reported clinical outcomes of individual studies using a random effect model or fixed effect model. The data was extracted and presented as Odd Ratio (OR) for recurrence rate and incidence of complications. Mean Difference (MD) was used for continuous variable (18). Statistical analysis was performed using Review Manager Version 5.1.2 (Cochrane Collaboration, Software Update, Oxford, United Kingdom). All *p*-values were two-sides, and *p*-value ≤0.05 was considered statistically significant. Statistical heterogeneity among the included clinical trials was evaluated using Higgins *I*^2^ statistic, which represents the percentage of total variation across studies. If the *I*^2^ statistic was less than 50%, the fixed effect model was used to analyze studies; Otherwise, the random effect model was used.

## Results

### Characteristics of included studies

**Figure 1:**
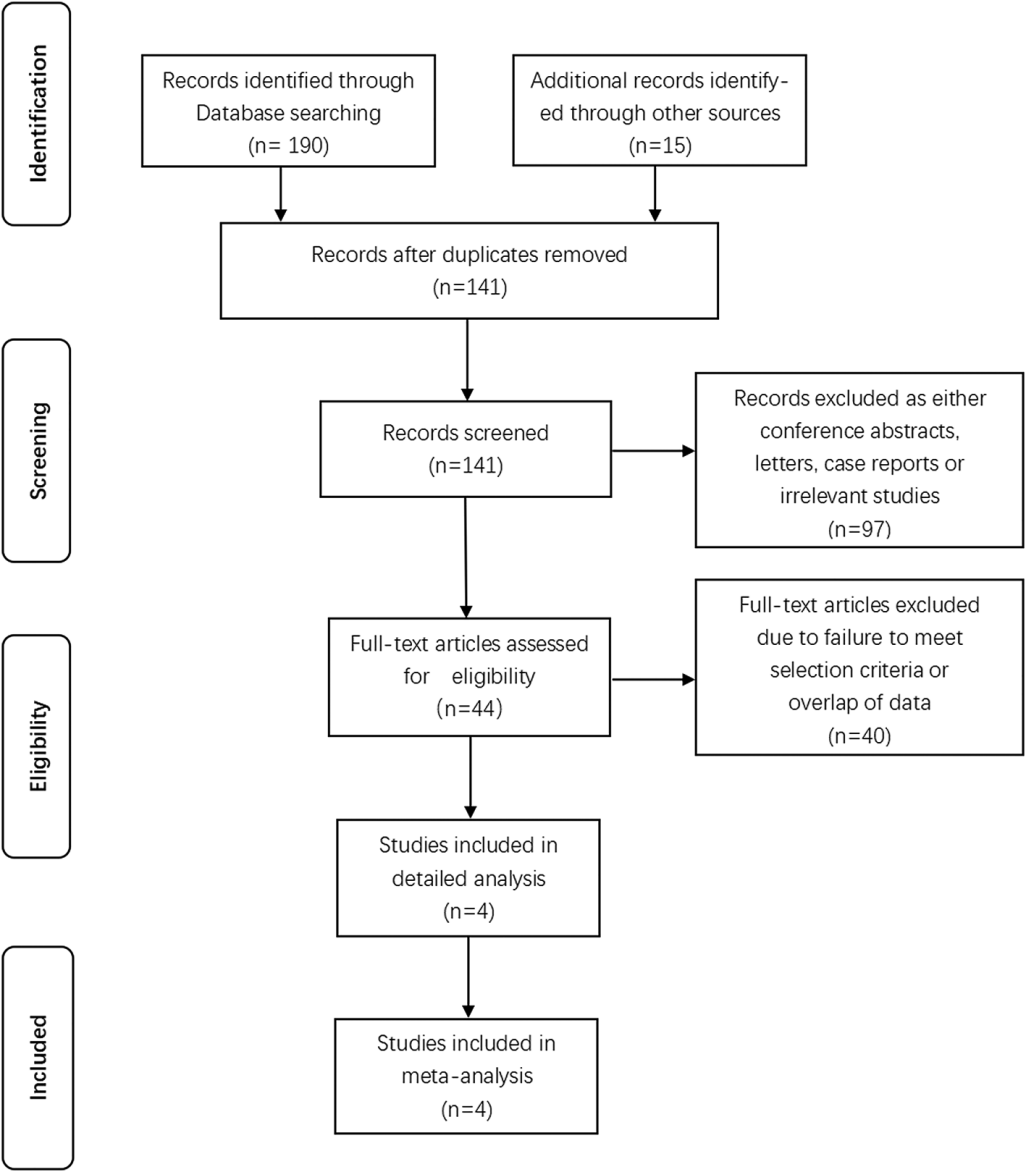
Preferred reporting items for systematic reviews and meta-analyses (PRISMA) flow diagram.

Initially we identified 205 studies after executing the search strategy. 141 records were remained after removing duplicated records. Further screening of titles and abstracts of remaining studies caused elimination of 97 unrelated records, leaving 44 studies. We reviewed full-text of remaining articles. Eventually, 4 studies that met inclusion criteria were absorbed into our study. A total of 1,229 participants were identified among which 325 patients who underwent subsegmentectomy and 904 patients who underwent segmentectomy. The identified studies were summarized in Figure 1 based on the PRISMA 2020 flow diagram (19). Table 1 showed the details of each trial, including publication year of the study, surgical procedure, and literature quality evaluation.

**Table 1:** Characteristics of identified studies.

Author	Year	Study design	Study period	Cases (*n*)	Operation	Stage	NOS
					SS/SG		
Chang et al. (13)	2019	Retro	2014.01–2018.12	278	57/221	IA	8
Chen et al. (20)	2020	Retro	2016.05–2017.12	227	93/134	IA	7
Chen et al. (21)	2021	Retro	2020.04–2020.12	367	107/260	IA	7
Kato et al. (14)	2021	Retro	2005.03–2020.05	357	68/289	IA	7

Retro, retrospective study; SS, subsegmentectomy; SG, segmentectomy; NOS, Newcastle-Ottawa Scale.

### Recurrence rate after operation

**Figure 2:**
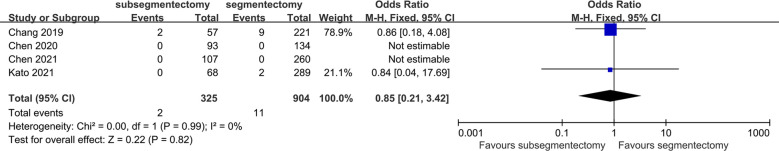
Forest plot: the recurrence rate of subsegmentectomy vs. segmentectomy.

The combined OR of the recurrence rate for subsegmentectomy and segmentectomy with NSCLC was 0.85 [95%CI (0.21, 3.42), *p *= 0.82]. The results showed that there was no significant discrepancy for recurrence rate between subsegmentectomy and segmentectomy (Figure 2).

### Blood loss

**Figure 3:**
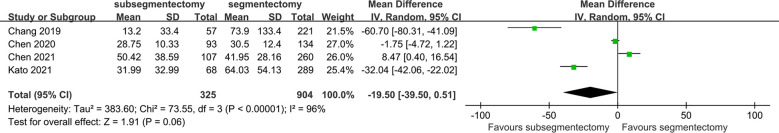
Forest plot: the blood loss of subsegmentectomy vs. segmentectomy.

The blood loss in the surgery was reported in 4 studies. The random effect model was applied in the research because of the significant heterogeneity. According to the above result, we concluded that the volume of blood loss of subsegmentectmoy was similar with that of segmentectomy [MD −19.50; 95%CI (−39.50, 0.51), *p *= 0.06] (Figure 3).

### Postoperative hospital stay

**Figure 4:**
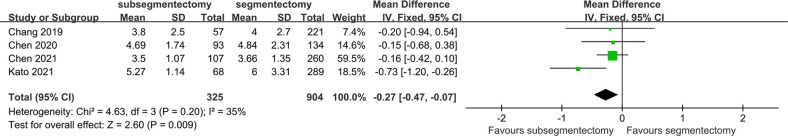
Forest plot: the postoperative hospital stay of subsegmentectomy vs. segmentectomy.

There were 4 studies identified in this comparison. The postoperative hospital stay of subsegmentectomy was significantly less than that of segmentectomy [MD 0.27; 95%CI (−0.47, −0.07), *p *= 0.009] (Figure 4).

### Duration of drainage

**Figure 5:**
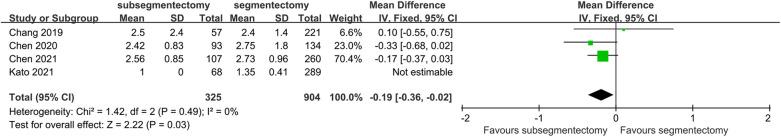
Forest plot: the duration of drainage of subsegmentectomy vs. segmentectomy.

Because the data in Hirohisa's study was not estimable, we excluded the study in this comparison. There was no significant heterogeneity in this comparison (*I*^2 ^< 50%, *p* > 0.1). Patients in the subsegmentectomy group had shorter duration of chest tube drainage than that patients in segmentectomy group [MD −0.19; 95%CI (−0.36, 0.02), *p* = 0.03] (Figure 5).

### Operation time

**Figure 6:**
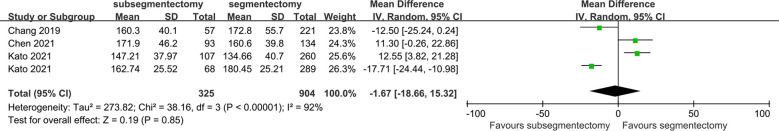
Forest plot: the operation time of subsegmentectomy vs. segmentectomy.

The operation time was reported in 4 studies. The random effect model was used to perform the meta-analysis because there was significant heterogeneity among trials (*I*^2^ = 90%, *p *< 0.1). Patients underwent subsegmentectomy had a comparable operation time compared with segmentectomy [MD −1.67; 95%CI (−18.66, 15.32), *p* = 0.85] (Figure 6).

### Incidence of complications

**Figure 7:**
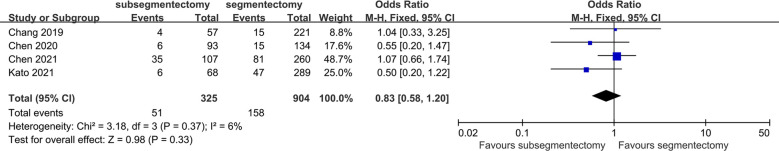
Forest plot: the postoperative complications of subsegmentectomy vs. segmentectomy.

There were 4 studies identified in this comparison. Comparative data among subsegmentectomy vs. segmentectomy demonstrated no significant difference for complications between surgical procedures [OR 0.83; 95%CI (0.58, 1.20), *p* = 0.33] (Figure 7).

## Sensitivity analysis and publication bias

**Figure 8:**
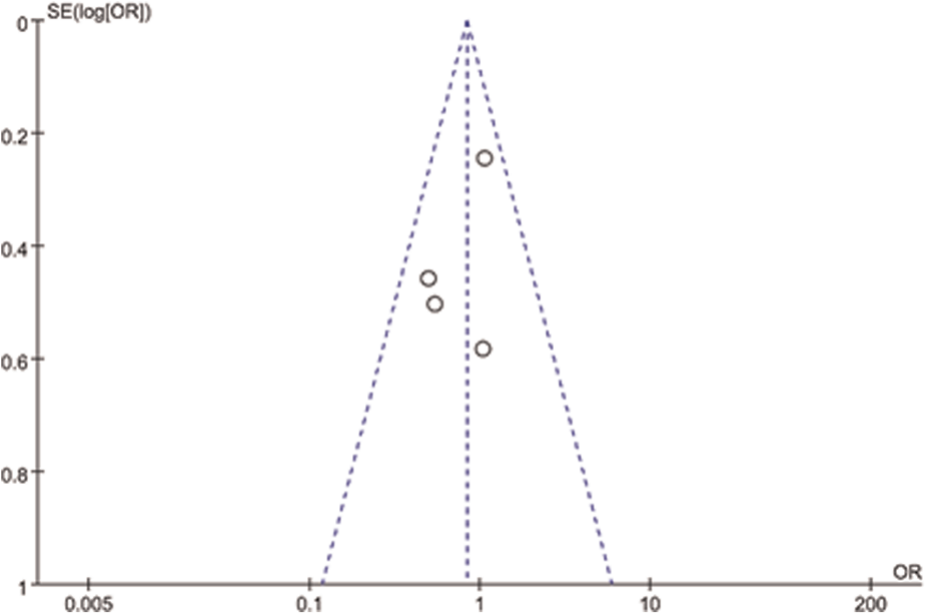
The funnel plot indicated a trend in publication bias and heterogeneity.

The results were similar when analysis was performed by fixed-effect model or random-effect model. A funnel plot was examined for asymmetry to determine publication bias and heterogeneity (Figure 8). The funnel plot was roughly symmetrical, suggesting that there was no publication bias and heterogeneity in this study.

## Discussion

Anatomic lobectomy has been generally considered as the standard surgical procedure for patients with early stage non-small cell lung cancer in the past several decades. Recently, with the development of surgical techniques and widespread use of HRCT, sublobar resection has gradually been a common treatment method for the operable patients (22–25). Wedge resection is used to treat pulmonary parenchymal peripheral nodule within 1/3 of visceral pleura. Segmentectomy is performed to treat deep and small sized lung nodule (26). Besides, wedge resection is considered as one of risk factors of postoperative recurrence, which may be insufficient to secure surgical margins (27). Although lobectomy or segmentectomy could completely resect lung cancer, it is assumed these procedures may lose extra lung tissues, resulting in loss of pulmonary function (28). In order to preserve more lung tissues and improve the pulmonary function, pulmonary subsegmentectomy with a smaller scope than segmentectomy has begun to be applied to the treatment of early stage NSCLC (16, 29–33). The surgical difficulty of subsegmentectomy is mainly reflected in the location of the lesion, the identification and separation of fine tissue structures, the protection of normal lung tissue, arteries and veins, the skill level of surgical operation and cooperation (21). Whether subsegmentectomy has comparative or even better clinical outcomes compared with segmentectomy remains controversial. The safety and efficacy of subsegmentectomy have not yet reached a unified conclusion. Consequently, it is necessary to summarize relevant studies to better understand the role of subsegmentectomy.

Due to the insufficient follow-up time and no specific time from completion of surgery to recurrence was provided in each study, we were only able to compare the perioperative clinical outcomes and count the frequency of recurrence in different procedures. In general, we found that subsegmentectomy showed comparable clinical outcomes compared with segmentectomy in this study. Specifically, both procedures showed similar recurrence rate in short term. Inadequate resection margin was considered one of the high-risk factors of local recurrence (34, 35). The result disclosed that subsegmentectomy could attain adequate resection margin like segmentectomy. The published studies have confirmed that there was no significant difference with regard to hospital stay between subsegmentectomy and segmentectomy (14, 16, 20, 21). Interestingly, when we compared postoperative hospital stay, we found that patients who underwent subsegmentectomy experienced shorter length of stay than those who underwent segmentectomy. From a clinical perspective, whether patients who underwent subsegmentectomy could benefit from shorter postoperative hospital stay needs further verification. Furthermore, the subsegmentectomy presented less duration of drainage than segmentectomy, suggesting less pleural effusion caused by subsegmentectomy. Generally, the indications for removal of closed thoracic drainage in patients with lung cancer after thoracic surgery were as follows: when the daily drainage volume was less than 100 ml/24 h, there was no air leakage for more than 24 h, the lung was completely dilated, the drainage tube could be removed (36). Compared with segmentectomy, subsegmentectomy had smaller resection scope and surgical wound, causing less chest drainage and rapid recovery of surgical wounds. When comparing the volume of blood loss, we found that there was no difference between the procedures, but a trend towards less blood loss in subsegmentectomy, which was consistent with previous study (13, 14). Results of researches about operation time remain controversial. Chen et al. considered that subsegmentectomy consumed more time than segmentectomy (21). On the contrary, two other studies disclosed that more time was needed for segmentectomy (13, 14). The random effect model was used to compare operation time because there was significant heterogeneity among studies. No statistically significant difference for operation time on subsegmentectomy vs. segmentectomy was observed in our study. The difference in results may be due to the surgeon's proficiency in surgery and the degree of structural variation of bronchi and blood vessels. Postoperative complications mainly included air leakage, hoarseness, pneumonia, dyspnea, chylothorax, and bronchopleural fistula (14, 16). Kato et al. reported there were few complications after subsegmentectomy (15, 37). Similarly, in our study perioperative complications of different surgical procedures showed no significant difference between subsegmentectomy and segmentectomy. There were few literatures regarding comparison of pulmonary function between two procedures. Yoshimoto et al. found that mean FEV1 was slightly higher in combined subsegmentectomy (CSS) than that in segmentectomy (0.05 ± 0.03 vs. 0.03 ± 0.02, *p* = 0.02) (13, 38). Chang et al. also thought subsegmentectomy could preserve more pulmonary function (13).

The difficulty of subsegmentectomy seems complex compared with segmentectomy. Actually, the learning curve of subsegmentectomy was considered to be equivalent to that of segmentectomy (13, 14). The application of 3D-CT was quite important to the performance of subsegmentectomy (15, 37). The undetectable pulmonary nodules with palpation or visualization could be successfully resected *via* 3D-CT simulation. It could identify variant pulmonary subsegmental arteries, intersegmental veins, bronchi, and decrease loss of blood as well as risk of operation. Moreover, 3D-CT cooperates with the inflation-deflation method which is common method to distinguish intersubsegmental plane (14).

Overall, subsegmentectomy showed the smaller excision extension and surgical wound. The patients underwent subsegmentectomy experienced short duration of drainage and postoperative hospital stay compared with segmentectomy. Similar operation time was required between subsegmentectomy and segmentectomy. Meanwhile, there was no difference in the two types of procedures with regard to the incidence of postoperative recurrence and postoperative complication. According to our study, subsegmentectomy could provide equivalent oncological results and as adequate resection margin as segmentectomy. All the results confirmed the safety and short-term efficacy of subsegmentectomy, which suggested that it was feasible for the performance of subsegmentectomy.

## Limitation

There were several limitations that should be noted. First, the analysis included retrospective studies which presented low evidence effectiveness. Second, the pulmonary function was not included in this analysis due to the small sample size of this variable. Furthermore, because of the short follow up period, the analysis result needs to be further confirmed through the randomized controlled trial which includes long term overall survival and disease-free survival.

## Conclusion

In conclusion, the present systematic analysis demonstrated that subsegmentectomy was feasible and could be an alternative treatment for the deep small sized lung cancer, which is less than 1.5 cm and GGO-dominant.

## Author contributions

LS, SL contributed to the conception and design of the study. XH and RJ contributed to systematic literature search and data extraction. WL, MZ and JW contributed to manuscript draft. TM, SZ, and SX contributed to editing and revision. All authors contributed to the article and approved the submitted version.
